# Evaluation of eritoran tetrasodium (E5564), a TLR4 antagonist, on the QTc interval in healthy subjects

**DOI:** 10.1186/cc9684

**Published:** 2011-03-11

**Authors:** CF Nagy, M Lynn, J Gogate

**Affiliations:** 1 Eisai, Inc., Woodcliff Lake, NJ, USA

## Introduction

Eritoran tetrasodium (E), a TLR4 antagonist, is currently being evaluated in phase 3 as a treatment for severe sepsis and has been well tolerated in clinical trials [1]. The primary objective of this study was to evaluate the effect of E on QTc in healthy subjects.

## Methods

This was a single 12-hour intravenous infusion, double-blind, placebo-comparator and active-comparator controlled, parallel-groupstudy. Subjects were randomized to: Arm A, E 2.3 mg/hour (a therapeutic(T) total dose of 28 mg); Arm B, E 7 mg/hour (a supratherapeutic (S) total dose of 84 mg); Arm C, placebo; or Arm D, placebo + moxifloxacin(M) 400 mg p.o. The primary outcome parameter was the placebo-corrected change from baseline in QTcF (ΔΔQTcF) based on the largest time-matched mean difference 10, 12, 14, 16, 18, 24, 36, and 48 hours after the start of infusion. Categorical and pharmacokinetic (PK)/pharmacodynamic (PD) evaluations were performed. Adverse events were reported.

## Results

Two hundred subjects (mean age 33.4 years; 81.5% male) were randomized. In the M group, the increase in QTcF from baseline (ΔQTcF) consistently exceeded placebo (maximum ΔΔQTcF 11.4 ms at 4 hours postdose). The lower bound of the one-sided 95% confidence limit was >5 ms at each time point between 2 and 8 hours postdose, indicating the study's sensitivity to demonstrate small QTc effects. The largest mean ΔΔQTcF for E was 2.1 ms (84 mg, 12 hours) and 1.6 ms (28 mg, 48 hours). The upper limit of the two-sided 90% CI (one-sided 95% CI) for the mean difference did not exceed 4.6 ms and all 90% CIs were inclusive of zero. No subject in either E group had a ΔQTcF exceeding 30 ms and only one subject in the E 84 mg group had a single QTcF >450 ms at 16 hours. QTcB, QTci, categorical, and PK/PD results all confirmed those from the primary analysis. There was no obvious correlation between QTcF and plasma E concentration. E 28 mg or 84 mg was safe and well tolerated, with mild headache most frequently reported in the placebo (9.6%) and E 28 mg (8.7%) groups, injection site hemorrhage in the E 84 mg group (6.1%), and nausea in the M group (3.8%).

## Conclusions

At either a T or S dose of E, a QTc effect exceeding 5 ms could be excluded. The upper bound of the 95% one-sided CI for ΔΔQTcF was <10 ms at both the S and T doses of E, indicating this is a negative thorough QT/QTc study.
